# Can the Abdominal Aortic Atherosclerotic Plaque Index Predict Functional Outcomes after Robot-Assisted Partial Nephrectomy?

**DOI:** 10.3390/diagnostics13213327

**Published:** 2023-10-27

**Authors:** Alessandro Veccia, Emanuele Serafin, Alessandro Tafuri, Sarah Malandra, Bogdan Maris, Giulia Tomelleri, Alessandro Spezia, Enrico Checcucci, Pietro Piazza, Severin Rodler, Loic Baekelandt, Karl-Friedrich Kowalewski, Ines Rivero Belenchon, Mark Taratkin, Stefano Puliatti, Pieter De Backer, Juan Gomez Rivas, Giovanni Enrico Cacciamani, Giulia Zamboni, Paolo Fiorini, Alessandro Antonelli

**Affiliations:** 1 Department of Urology, Azienda Ospedaliera Universitaria Integrata Verona, 37126 Verona, Italyalessandro_antonelli@me.com (A.A.); 2 Department of Urology, Vito Fazzi Hospital, 73100 Lecce, Italy; 3 Department of Surgery, Dentistry, Pediatrics and Gynecology, University of Verona, Azienda Ospedaliera Universitaria Integrata (AOUI) Verona, 37126 Verona, Italygiulia.zamboni@univr.it (G.Z.); 4 Department of Computer Science, University of Verona, 37126 Verona, Italy; bogdan.maris@univr.it (B.M.); paolo.fiorini@univr.it (P.F.); 5 Department of Radiology, Azienda Ospedaliera Universitaria Integrata Verona, 37126 Verona, Italy; 6 Department of Surgery, Candiolo Cancer Institute, FPO-IRCCS, 10060 Turin, Italy; 7 Division of Urology, IRCCS Azienda Ospedaliero-Universitaria di Bologna, 40138 Bologna, Italy; 8 Department of Urology, LMU University Hospital, LMU Munich, 81377 Munich, Germany; 9 Department of Urology, University Hospitals Leuven, 3000 Leuven, Belgium; 10 Department of Urology, University Medical Center Mannheim, University of Heidelberg, 69117 Mannheim, Germany; 11 Urology and Nephrology Department, Virgen del Rocío University Hospital, Manuel Siurot s/n, 41013 Seville, Spain; ines.rivero.belenchon@gmail.com; 12 Institute for Urology and Reproductive Health, Sechenov University, 119992 Moscow, Russia; marktaratkin@gmail.com; 13 Department of Urology, University of Modena and Reggio Emilia, 41126 Modena, Italy; stefanopuliatti@gmail.com; 14 ORSI Academy, 9090 Melle, Belgium; 15 Department of Urology, Hospital Clinico San Carlos, 28040 Madrid, Spain; juangomezr@gmail.com; 16 USC Institute of Urology, University of Southern California, Los Angeles, CA 90007, USA

**Keywords:** robot-assisted partial nephrectomy, abdominal aortic plaque atherosclerotic index, chronic kidney disease

## Abstract

This study aims to evaluate the abdominal aortic atherosclerotic plaque index (API)’s predictive role in patients with pre-operatively or post-operatively developed chronic kidney disease (CKD) treated with robot-assisted partial nephrectomy (RAPN) for renal cell carcinoma (RCC). One hundred and eighty-three patients (134 with no pre- and post-operative CKD (no CKD) and 49 with persistent or post-operative CKD development (post-op CKD)) who underwent RAPN between January 2019 and January 2022 were deemed eligible for the analysis. The API was calculated using dedicated software by assessing the ratio between the CT scan atherosclerotic plaque volume and the abdominal aortic volume. The ROC regression model demonstrated the influence of API on CKD development, with an increasing effect according to its value (coefficient 0.13; 95% CI 0.04–0.23; *p* = 0.006). The Model 1 multivariable analysis of the predictors of post-op CKD found that the following are independently associated with post-op CKD: Charlson Comorbidity Index (OR 1.31; *p* = 0.01), last follow-up (FU) Δ%eGFR (OR 0.95; *p* < 0.01), and API ≥ 10 (OR 25.4; *p* = 0.01). Model 2 showed API ≥ 10 as the only factor associated with CKD development (OR 25.2; *p* = 0.04). The median follow-up was 22 months. Our results demonstrate API to be a strong predictor of post-operative CKD, allowing the surgeon to tailor the best treatment for each patient, especially in those who might be at higher risk of CKD.

## 1. Introduction

Partial nephrectomy (PN) represents the standard of care for the treatment of localized renal cell carcinoma (RCC) whenever feasible [1] and its use has spread with the advent of robotics which reduces intra- and postoperative morbidity [2,3]. Besides providing optimal surgical results, PN shows oncological outcomes comparable to radical nephrectomy, while protecting cardiological and renal function [4,5]. Kidney function preservation is paramount in such cases, and several unmodifiable (patient age, baseline kidney function, comorbidities) and modifiable (ischemia technique, resection technique, and renorraphy technique) factors are involved in this process [6]. Ischemia time has been one of the most assessed factors for decades and several tools, such as near-infrared fluorescence [7], as well as surgical techniques (selective clamping, early unclamping, and zero ischemia), have been assessed and adopted to limit its duration [8]. However, the CLOCK trial [9] shows that, in patients with two kidneys and a normal glomerular filtration rate (GFR), a modifiable factor such as ischemia time does not impact functional outcomes when the arterial clamping time is limited [10]. The overcoming of the “tick-tock dance” [11] led to the evaluation of patients’ pre-operative medical status rather than ischemia being used as a predictor of post-operative kidney function. Among the comorbidities responsible for post-nephron-sparing renal function impairment, hypertension, diabetes, and chronic kidney disease (CKD) seem to be the most involved [12]. These are among the etiological factors of atherosclerosis, which has a deleterious effect on hemodynamics, especially when affecting large vessels [13]. These data have been confirmed for subclinical renal disease related to renal artery calcification [14].

The Aortic and Renal Arteries Calcium Score, a radiological score computing the calcification of arterial vessels, found that the Aortic Calcium Score is an independent predictor of chronic kidney disease after nephron-sparing surgery (NSS) (odds ratio (OR) 4.07; *p* = 0.029) [15]. The abdominal aortic atherosclerotic plaque index (API) assesses the ratio between the plaque volume and the total volume of the abdominal aorta, and represents an easy and valuable tool for patient counseling and the evaluation of surgical strategy [16].

This study aims to evaluate the predictive role of API in patients with pre-operatively or post-operatively developed CKD treated with robot-assisted partial nephrectomy (RAPN) for RCC.

## 2. Material and Methods

The prospectively maintained RCC Institutional database was retrospectively queried to retrieve data on RAPN for RCC. Of 643 patients, 183 patients (134 with no pre- and post-operative CKD (no CKD) and 49 with persistent or post-operative CKD development (post-op CKD)) who underwent RAPN between January 2019 and January 2022 were deemed eligible for inclusion in the analysis. Those lacking FU data and available CT imaging for the measurements were discarded. All the procedures were performed by two experienced surgeons far beyond the learning curve.

### 2.1. API Evaluation

All patients had available contrast-enhanced CT scans with slices at least 2 mm in thickness.

The detailed measurement of API has been described elsewhere [16].

A user-friendly radiological interface was developed to segment the aorta, the plaque and the computation of the API. An upgraded algorithm was integrated into ©MeVisLab (©MeVis Medical Solutions AG, Bremen, Germany) [17], a powerful modular framework focused on medical imaging that allows imaging processing and development. It allows quantitative segmentation, registration, and volumetric assessment, as well as quantitative morphological and functional analysis. The API evaluation consisted of three phases.

During the first step, the user identifies the region of interest (ROI) on the CT scan in three different projections (transversal, sagittal, and coronal). For study purposes, the ROI was the aorta from its origin until the iliac vessels. This ROI was set as the standard and applied to all the assessed CT scans.

The second step is focused on aortic contouring at different levels (e.g., 1 every 10 slides), which are used by the software for curve interpolation. After this process, the aorta is completely highlighted, and a narrower ROI is created. The trade-off between manual selection and curve interpolation helps to speed up the segmentation process or improve its quality. Once the optimal selection is achieved, a new ROI is created to isolate the aorta from other anatomical structures, to be able to move to the next step of the aorta and plaque segmentation.

The final step is characterized by the automatic segmentation of the aorta and its atherosclerotic plaque through a growing algorithm. By setting two Hounsfield unit thresholds, the lower for the aorta and the higher for the plaque, no overlap occurs during the automatic segmentation. Finally, the API is computed automatically from the 3D model.

A mean time of 10 min is required for each API determination.

### 2.2. Variable Definition

Patients’ baseline characteristics (age at surgery, gender, body mass index (BMI), Charlson Comorbidity Index (CCI), ASA (American Society of Anesthesiology) Score, hypertension, coronary artery disease, diabetes, pulmonary disease, pre-operative eGFR according to CKD-EPI, pre-operative Hb, pre-operative albumin, IPA), tumor features (tumor side, tumor dimension, RENAL score, cTNM), operative data (artery clamping, ischemia time, estimated blood loss (EBL), length of stay (LOS), major complications according to Clavien–Dindo), pathological outcomes (tumor dimension, histology, pTNM, positive surgical margins (PSM)), and functional outcomes (pre-operative, 6-month, 12-month, 24-month, and last follow-up (FU) eGFR) were collected within a dedicated password coded dataset.

The Δ%eGFR variation at each time point (discharge, 6-month, 12-month, 24-month, and last FU) was calculated according to the following formula:

[(eGFR _last time point_ − eGFR _preoperative_)/eGFR _preoperative_] × 100.

Based on histology, all patients were followed up according to current EAU Guidelines [1]. A 6-month, and then annual, contrast-enhanced total body CT/MRI or chest X-ray and abdominal ultrasound with functional work-up were prescribed.

### 2.3. Endpoint

The primary endpoint was to evaluate the association of API with CKD development or worsening.

### 2.4. Statistical Analysis

The statistical analysis was conducted following the Guidelines for the Reporting of Statistics for Clinical Research in Urology [18].

A graphical assessment of data distribution was performed to evaluate parametric and non-parametric test applications. The mean interquartile range (IQR) as well as frequencies and proportions were adopted to report the continuous and dichotomous variables, respectively. The Mann–Whitney U test was used to evaluate the differences among continuous variables, whereas Fisher’s exact test was indicated for the dichotomous variables.

A scatter plot graph was designed to evaluate the graphical distribution and relationship between API values and the last FU eGFR.

A ROC regression analysis was performed to fit the maximum likelihood model to estimate the effect of the Δ%eGFR classifier on CKD development, assuming the extra effect of API on CKD development at different thresholds (10-15-20), accounting for a control population with hypertension, coronary artery disease and diabetes.

To evaluate the OR and 95% confidence interval (CI) of CKD development, two univariable and multivariable logistic regression models were built. The first one included the baseline characteristics of patients (ASA Score, CCI, pre-operative eGFR, and API categorized as <10 and ≥10), and the second one included perioperative data (RENAL score, ischemia time, Clavien–Dindo, and API categorized as <10 and ≥10) as covariates.

All the analyses were performed using Stata^®^ 17.0 (StataCorp 2017. Stata Statistical Software: release 15. StataCorp LLC, College Station, TX, USA) and the following syntax was adopted: *histogram*, *tabstat, tabulate, exact, two-way scatter, rocreg, rocregplot,* and *logistic*. All tests were two-sided and statistical significance was set at *p* ≤ 0.05.

## 3. Results

Compared to the no CKD group, the patients in the post-op CKD group were older (72 vs. 63 years; *p* < 0.001), had a worse CCI (*p* < 0.001) and ASA Score (*p* = 0.0002), and a higher rate of hypertension (75.5 vs. 48.5%; *p* = 0.001) and diabetes (36.7 vs. 11.2%; *p* < 0.001). The post-op CKD group had higher API scores (2.5 vs. 0.25; *p* < 0.0001) compared to the no CKD group. On the contrary, no statistically significant difference was detected in terms of tumor features (Table 1). The median FU length was of 22 months (IQR 12-33).

In terms of operative data, the patients in the post-op CKD group recorded higher EBL (200 mL vs. 100 mL; *p* = 0.004), but no statistically significant difference was found in complications ≥3 according to Clavien–Dindo. No statistically significant differences were found regarding pathological outcomes (Table 2).

The functional outcomes demonstrated a worse eGFR at each time point (discharge, 6-, 12-, 24-month, and last FU) for the post-op CKD group (*p* < 0.001), whereas Δ%eGFR was significantly lower at the last FU only for the post-op CKD group (-12 vs. -2.6; *p* = 0.0001) (Table 3).

**Figure 1:**
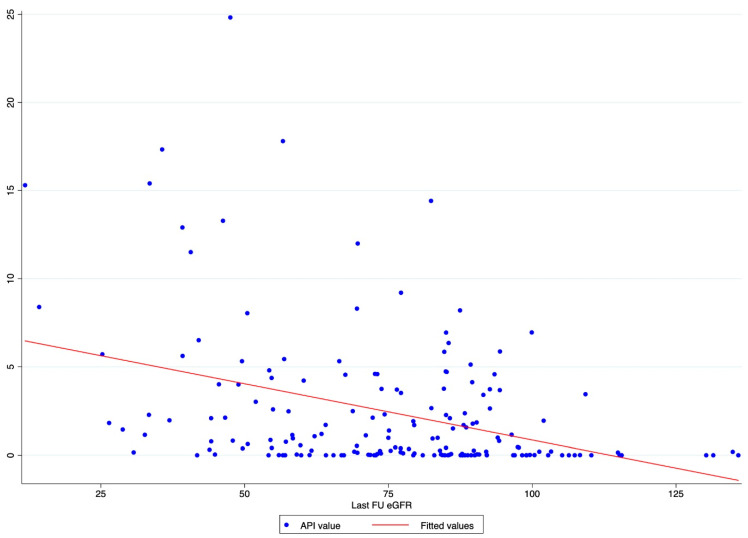
Scatter plot graph of API value distribution showing an inverse relationship between API and the last FU eGFR.

**Figure 2:**
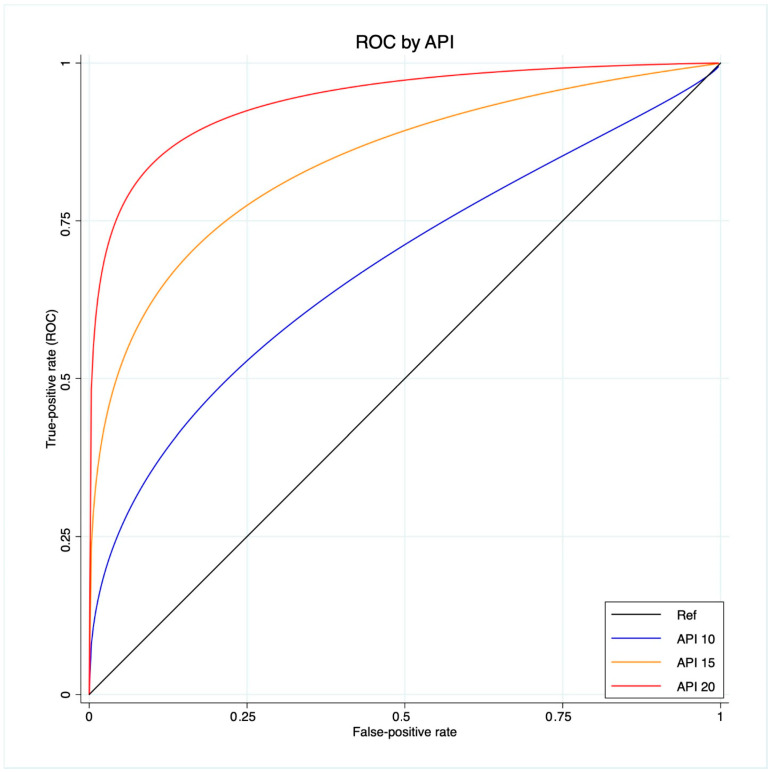
ROC regression model that fits the maximum likelihood model to estimate the effect of the Δ%eGFR classifier on CKD development, assuming the extra effect of API at different thresholds (10-15-20) on CKD development, accounting for a control population with hypertension, coronary artery disease and diabetes.

The scatter plot graph demonstrates an inverse relationship between API and the last FU eGFR (Figure 1). The ROC regression model demonstrated the influence of API on CKD development with an increasing effect according to its value (coefficient 0.13; 95% CI 0.04-0.23; *p* = 0.006) (Figure 2). The AUC values were as follows: API 10 AUC 0.57, API 15 AUC 0.77, API 20 AUC 0.84.

The Model 1 multivariable analysis of the predictors of post-op CKD found CCI (OR 1.27; 95% CI 1.04–1.56; *p* = 0.02) and API ≥ 10 (OR 15.8; 95% CI 2.20–113.4; *p* = 0.01) to be independently associated with post-op CKD. Model 2 showed API ≥ 10 as the only factor associated with CKD development (OR 25.2; 95%CI 1.15–549.06; *p* = 0.04) (Table 4).

## 4. Discussion

The abdominal aortic plaque atherosclerotic index is a novel measure to predict functional outcomes after PN. A previous analysis of PN independently demonstrated this index to be a valuable tool to evaluate eGFR variation after surgery [16], independent of the surgical approach. Nevertheless, the authors focused on the potentiality of this tool for evaluating short-term kidney function, and the results might have been impaired by the inclusion of different surgical approaches. In this paper, we have presented the application of this assessment method within a large cohort of RAPN, leading to several interesting findings.

Our analysis showed API to be associated with CKD after RAPN, with an inverse relation with the post-operative eGFR value. Indeed, the scatter plot showed the higher the API value, the lower the last FU eGFR (Figure 1). This finding was further confirmed via the ROC regression model, which underlined that an increasing API influenced CKD development and Δ%eGFR at the last FU. This was especially evident above an API value of ten (Figure 2). These findings corroborate the well-established evidence that sustains that the presence of atherosclerotic plaques predisposes patients to kidney function impairment, in addition to cardiovascular events [12]. Furthermore, the altered blood flow exposes the endothelium to different shear stress, which seems to be associated with the development of atherosclerosis. Indeed, in the case of vascular lumen sub-stenosis, high shear stress develops in the restriction site, but low laminar and low oscillatory stresses are recorded upstream and downstream, respectively [19]. These hemodynamic changes induce the endothelial cells to an atherosclerotic-prone behavior, where low shear stress is recorded [20]. This hemodynamic impairment, in combination with aging, hypertension, and other predisposing factors, determines arterial calcification [21]. The latter allows the evaluation of an atherosclerotic plaque via CT scan, and the Arterial Calcium Score, a cardiologically conceived tool, has been largely investigated in cardiology to assess several clinical outcomes [22].

Recently, Akarken et al. evaluated the Aortic and Renal Artery Calcium Score, which accounts for the atherosclerotic plaque volume alone, as a means to predict functional outcomes after kidney surgery for renal tumors. Within a retrospective cohort of 302 patients, 16.6% had post-operative CKD, and a median aortic and renal artery calcium score of 950 (0–10038) and 0 (0–3009), respectively, which was higher compared to the no CKD group (*p* < 0.001). The authors identified a threshold of 134 as a clinically meaningful value, and patients with a higher score had an approximately 4-fold chance of developing CKD (*p* = 0.029) [15]. On the contrary, the API, which is the ratio between atherosclerotic plaque and the aorta lumen volume, can provide indirect data on aortic obstruction. Indeed, a value of 0 does not indicate the presence or not of an atherosclerotic plaque, but rather the absence of an imbalance between the plaque and the vessel lumen. An increasing API value represents a progressive reduction in aortic patency. Accordingly, in the multivariable analysis, we did find that patients with API ≥ 10 had about a 25-fold chance of post-op CKD compared to those with lower API values (Table 4). Additionally, API, CCI, and the last FU Δ%eGFR indicated an association with post-operative CKD. Our findings are in line with those of Xiong L et al., who recently raised the point that “within the context of conventional, limited durations of ischemia, histologic deterioration of preserved parenchyma after PN appears to be primarily due to pre-existing medical comorbidities rather than ischemia” [12]. Indeed, the authors retrospectively reviewed 65 patients treated first with PN for renal cancer, and subsequently treated with radical nephrectomy for tumor recurrence. Using the CKD score, which is a summary of glomerular/tubular/interstitial/vascular status, the authors found that, during the pathological specimen evaluation, the score increase was mainly due to pre-existing hypertension, diabetes, and CKD.

Data from the CLOCK randomized trial demonstrated both no impact of ischemia time on renal function in the context of two kidneys and normal function [10], and the negligible clinical effects of ischemia above the threshold of 10 min [23], in those patients undergoing RAPN. However, this was a multicenter randomized trial involving very experienced surgeons, and the results might be different in a common clinical scenario. A meta-analysis of comparative studies about off- vs. on-clamp PN demonstrated that this procedure is still dedicated to small renal masses and that, under such conditions, there is no difference between the two procedures. Indeed, the functional outcomes assessed as eGFR at early postoperative, 3-month, 6-month, and the last available FU were not statistically different [24]. In our cohort, the ischemia time was longer in the post-op CKD group, but still under the well-known threshold time impairing kidney function. Therefore, we built a multivariable model to assess whether the RENAL Score, ischemia time, and postoperative complications as covariates might balance the API value. Once again, API ≥ 10 was the only independent predictor of post-operative CKD (OR 25.20; *p* = 0.04). The latter result supports kidney function preservation as a multifactorial condition mainly due to the patient’s medical history and surgical factors. Recent evidence demonstrates that one of the main determinants of functional recovery after NSS is renal parenchyma preservation. Munoz-Lopez, C. et al. evaluated 670 patients who underwent PN with warm, cold, and zero ischemia. In this retrospective analysis, functional recovery correlated strongly with preserved parenchymal volume (r = 0.83; *p* < 0.01). On the contrary, the median (IQR) recovery from arterial management was similar across the three different techniques (warm 96% (90–102%), cold 95% (89–101%), and zero ischemia 97% (91–102%)) [25]. According to these data, the attention is moving more and more toward other factors rather than focusing solely on ischemia. Notwithstanding this, the patients involved in our analysis had mostly low–intermediate complexity tumors not requiring a prolonged ischemia time and allowing the preservation of the majority of the renal parenchyma. Aside from this limitation, there are others that might mean these findings are not replicable in another context. Indeed, this is a single tertiary academic center cohort that includes patients treated by highly experienced surgeons far beyond the learning curve [26]. Moreover, the retrospective nature of the dataset, even if prospectively maintained, exposes the study to intrinsic bias. Indeed, despite our data representing a baseline for further investigation, the application of this index and the low number of patients in the post-op CKD group might have made our ORs higher than expected. In addition, the definition of CKD development and persistence is based on eGFR only and does not consider other important factors, such as albuminuria, which might have required the groups to have been assessed differently. The FU length might be not enough to confirm our results. Indeed, according to previous long-term evidence, the FU time should be at least 60 months [27]. Lastly, the measurements were performed by urology and radiology residents, and some discrepancies might be due to the different expertise in viewing CT imaging. Unfortunately, we were not able to stratify the results according to different levels of experience because of the small sample size. Despite these drawbacks, several tools are emerging both for preoperative planning and the prediction of patient outcomes [28], and API seems to be promising. With the era of artificial intelligence upon us [29,30], this imaging assessment instrument could be automated, making its application easier and faster.

## 5. Conclusions

The abdominal aortic plaque atherosclerotic index represents an easy and useful tool to predict the risk of CKD disease in patients undergoing RAPN. However, API might not be easy to use in a routine practice requiring dedicated software that might not be available in all centers and on all computers. Our results show that API is a strong predictor of post-operative CKD, allowing the surgeon to tailor the best treatment for each patient, especially in those who might be at higher risk of CKD. Despite these promising results, further results from larger, multicenter, and variegate cohorts will be necessary to confirm these data.

## Figures and Tables

**Table 1:** Baseline characteristics of patients and tumor characteristics.

Variable	No CKD (134)	Post-op CKD (49)	*p*-Value
**Baseline characteristics**			
Age	63 (54-71)	72 (67-77)	**<0.001**
Gender (male)	86 (64.2)	30 (61.2)	0.73
BMI	25.6 (23.4-28.8)	26.7 (24.2-29)	0.27
CCI	4 (2-5)	5 (4-7)	**<0.001**
ASA Score	2 (2-2)	2 (2-3)	**0.0002**
Hypertension	65 (48.5)	37 (75.5)	**0.001**
Pulmonary disease	17 (12.7)	8 (16.3)	0.63
Coronary artery disease	13 (9.7)	10 (20.4)	0.08
Diabetes	15 (11.2)	18 (36.7)	**<0.001**
Pre-operative Hb	14.4 (13.4-5.3)	13.4 (12.4-4.9)	**0.003**
Pre-operative eGFR	88.5 (78.9-97.8)	56 (45.7-70.6)	**<0.001**
Pre-operative albumin	42.4 (39.3-45)	41.9 (38.2-45)	0.40
API	0.25 (0-2.3)	2.1 (0.6-5.6)	**0.0001**
**Tumor characteristics**			
Tumor side left	70 (52.2)	22 (44.9)	0.40
Tumor dimension	3 (2.3-4)	3 (2.2-4)	0.81
RENAL Score	7 (5-8)	7 (5-8)	0.71
cT			0.92
1a	103 (76.9)	39 (79.6)	
1b	23 (17.6)	9 (18.4)	
2a	4 (3)	-	
2b	1 (0.7)	-	
3a	3 (2.1)	1 (2)	

BMI = body mass index; CCI = Charlson Comorbidity Index; ASA = American Society Anesthesiology; API = abdominal aortic atherosclerotic plaque index. Bold numbers are statistically significant values.

**Table 2:** Operative and pathological outcomes.

Variable	No CKD (134)	Post-op CKD (49)	*p*-Value
**Operative data**			
On-clamp	53 (39.8)	15 (30.6)	0.30
Ischemia time	14 (11-19)	19 (10-24.5)	0.14
EBL	100 (10-250)	200 (150-350)	**0.004**
LOS	4 (3-5)	4 (3-5)	0.07
Clavien–Dindo ≥ 3			0.36
3a	4/130 (3.1)	2/48 (4.2)	
3b	1/130 (0.8)	2/48 (4.2)	
4a	-	1/48 (2.1)	
**Pathological outcomes**			
Tumor dimension	3 (2.3-4)	3.5 (2.7-4.8)	0.13
Histology			0.15
cRCC	65/133 (48.9)	27 (55.1)	
pRCC	19/133 (14.3)	11 (22.5)	
chRCC	9/133 (6.8)	1 (4.1)	
oRCC	11/133 (8.2)	-	
Oncocytoma	17/133 (12.8)	5 (10.2)	
Angiomyolipoma	7/133 (5.3)	1 (2)	
Other	5/133 (3.7)	3 (6.1)	
pT			0.63
1a	100/128 (78.1)	35/47 (74.5)	
1b	20/128 (15.6)	10/47 (21.3)	
2a	1/128 (0.8)	1/47 (2.1)	
2b	2/128 (1.6)	1/47 (2.1)	
3a	5/128 (3.9)	-	
PSM	10/125 (8)	4/44 (9.1)	0.76

EBL = estimated blood loss; LOS = length of stay; PSM = positive surgical margins. Bold numbers are statistically significant values.

**Table 3:** Functional outcomes.

Variable	No CKD (134)	Post-op CKD (49)	*p*-Value
eGFR at discharge	88.5 (77.8-98.7)	54.5 (44.5-59.7)	**<0.001**
Δ%eGFR at discharge	-0.5 (-6.7; 5.7)	-3.7 (-17.6; 11.9)	0.44
6-month eGFR	84.7 (73.4-89.7)	45.4 (34.7-59.9)	**<0.001**
6-month Δ%eGFR	-4.9 (-10.5; 0.5)	-5.9 (-20.4; 8.7)	0.64
12-month eGFR	84.5 (75.7-94.4)	52.2 (36.8; 61)	**<0.001**
12-month Δ%eGFR	-4.2 (-11.1; 2.4)	-9.2 (-22.2; 0.41)	0.10
24-month eGFR	81.4 (74.5-92.2)	52.3 (39-58.4)	**<0.001**
24-month Δ%eGFR	-1.48 (-13.4; 2.0)	-5.51 (-19.4; 12.2)	0.77
Last FU eGFR	85.7 (76.5-94.2)	47.9 (39.2-54.9)	**<0.001**
Last FU Δ%eGFR	-2.6 (-10.2; 4.5)	-12 (-32; -2.4)	**0.0001**

eGFR = estimated glomerular filtration rate; FU = follow-up. Bold numbers are statistically significant values.

**Table 4:** Univariable and multivariable analysis of the predictors of CKD development.

	Univariable Analysis			Multivariable Analysis		
Variable	OR	95%CI	*p*-Value	OR	95%CI	*p*-Value
**MODEL 1** ^†^						
ASA Score	3.50	1.72–6.70	**<0.01**	1.74	0.77–3.89	0.20
CCI	1.42	1.19–1.69	**<0.01**	1.27	1.04–1.56	**0.02**
Pre–operative eGFR	0.98	0.96–0.01	0.28	0.98	0.96–1.01	0.38
API						
0	Ref			Ref		
<10	2.96	1.06–8.22	**0.04**	2.22	0.68–7.25	0.18
≥10	28	4.6–171.22	**<0.01**	15.8	2.20–113.4	**0.01**
**MODEL 2** ^‡^						
RENAL Score	0.97	0.82–1.15	0.76	0.85	0.58–1.24	0.39
Ischemia time	1.06	0.99–1.14	0.09	0.76	0.32–1.80	0.53
Post–operative complications	1.24	0.91–1.71	0.17	1.07	0.99–1.17	0.08
API						
0	Ref			Ref		
<10	2.96	1.06–8.22	**0.04**	2.54	0.46–13.86	0.28
≥10	28	4.6–171.22	**<0.01**	25.20	1.15–549.06	**0.04**

ASA = American Society of Anesthesiology; CCI = Charlson Comorbidity Index; eGFR = estimated glomerular filtration rate; API = abdominal aortic atherosclerotic plaque index, ^†^ r^2^ = 0.25, ^‡^ r^2^ = 0.12. Bold numbers are statistically significant values.

## Data Availability

Data available on request.
